# Structural and Optical Properties of Nanoscale Galinobisuitite Thin Films

**DOI:** 10.3390/ijms15021842

**Published:** 2014-01-27

**Authors:** Omar H. Abd-Elkader, N. M. Deraz

**Affiliations:** 1 Department of Zoology, Science College, King Saud University, P.O. Box 2455, Riyadh 11451, Saudi Arabia; 2 Electron Microscope and Thin Films Department, National Research Center (NRC), El-Behooth Street, Dokki, Cairo 12622, Egypt; 3 Laboratory of Surface Chemistry and Catalysis, Physical Chemistry Department, National Research Center, Dokki, Cairo 12622, Egypt, E-Mail: nmderaz@yahoo.com

**Keywords:** Galinobisuitite (Bi_2_S_3_)(PbS) thin film, CBD, nanoscale structural, XRD, AAS, optical band gap

## Abstract

Galinobisuitite thin films of (Bi_2_S_3_)(PbS) were prepared using the chemical bath deposition technique (CBD). Thin films were prepared by a modified chemical deposition process by allowing the triethanolamine (TEA) complex of Bi^3+^ and Pb^2+^ to react with S^2−^ ions, which are released slowly by the dissociation of the thiourea (TU) solution. The films are polycrystalline and the average crystallite size is 35 nm. The composition of the films was measured using the atomic absorption spectroscopy (AAS) technique. The films are very adherent to the substrates. The crystal structure of Galinobisuitite thin films was calculated by using the X-ray diffraction (XRD) technique. The surface morphology and roughness of the films were studied using scanning electron microscopes (SEM), transmission electron microscopes (TEM) and stylus profilers respectively. The optical band gaps of the films were estimated from optical measurements.

## 1. Introduction

Groups IV–VI and V–VI compounds are of continuing interest for many potential and actual applications in heterojunctions, solar cells, photo detectors, thermoelectric devices, photo electrochemical devices, and IR detectors. Bismuthinite and Galena in the form of thin films are promising materials for photoelectronic devices and IR detectors as their energy band gap is between 1.92 and 0.4 eV [1,2]. Direct band gap semiconductors with band gaps in the range 1.2 to 1.7 eV are well suited to convert light into electricity. In this respect, Galinobisuitite (Bi_2_S_3_)(PbS) seems to be a promising material.

In the present work, the most favorable results are obtained with thin films of Galinobisuitite (Bi_2_S_3_)(PbS) formed by the chemical bath deposition (CBD) method. Capping agents stabilize the nanoparticles and prevent them from aggregating. Polymers are good stabilizing agents because they can cover a larger surface area of the nanoparticles. The optical band gap values of nanoparticles are changed by increasing the molar concentration of the capping agent. These values exhibit a blueshift in E_g_, which is related to the decrease in the size of the particles and to the nanoparticles reaching the quantum confinement limit, the emission spectra of semiconductors strongly depend on the energy band structure of the material. The dependence of the band gap and its tuning, which relies on the size of the nanoparticles (quantum dots) and on the carrier concentrations, is important because of its surprising electronic, optical, and physical properties [3,4]. The structural, morphological, and optical properties of the Bi–Pb–S system were investigated, since various compositions between the stable Bi_2_S_3_ and PbS phases can be obtained. The differences in the crystallographic structure (orthorhombic for Bi_2_S_3_ and cubic for PbS) and the absorption edges (in the visible region for Bi_2_S_3_ and near infrared for PbS) constitute a sufficient argument to promote structural and optical investigations [5]. The material obtained was characterized by X-ray diffraction (XRD) and optical measurements, and the results are discussed in connection with the crystal structure of the film. To the best of our knowledge, no report on the structural and optical properties of polycrystalline stoichiometric films of Galinobisuitite (Bi_2_S_3_)(PbS), prepared by CBD, has been published.

## 2. Results and Discussion

### 2.1. Structural Analysis

Fifteen mineral species were reported having compositions within the (Bi_2_S_3_)*_m_*(PbS)*_n_* system. These lie close to (Bi_2_S_3_)(PbS) and are listed together with their reported compositions [6].

#### 2.1.1. Crystal Structure of Bi_2_S_3_

**Figure 1.**
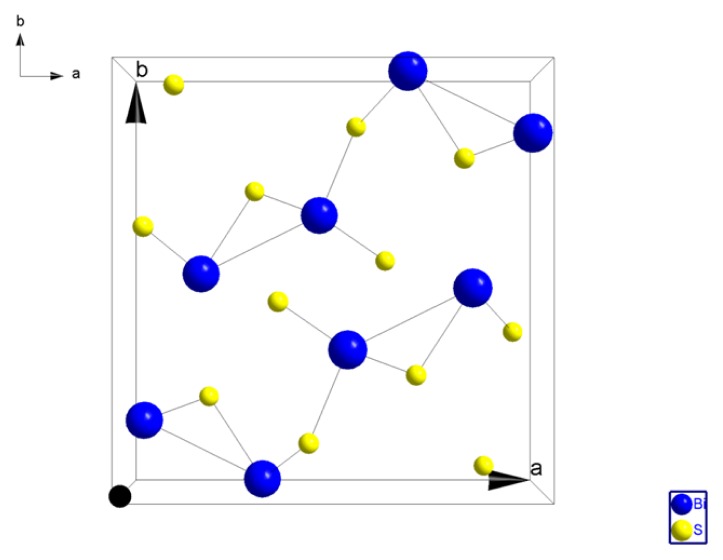
Structure of (Bi_2_S_3_).

Bi_2_S_3_ has an orthorhombic lattice with four formula units per unit cell, beyond these, there are three or four additional neighbors at distances that are slightly larger as shown as in Figure 1.

This structure can be considered as being made up of puckered sheets or planes of stoichiometric composition perpendicular to the (001) direction. The bonding between these sheets is considerably weaker than that within the sheets [7]. This suggests that cleavage would take place on (010) planes, and crystal growth in the (001) direction, these properties were found to belong these compounds [8].

#### 2.1.2. Crystal Structure of PbS

**Figure 2.**
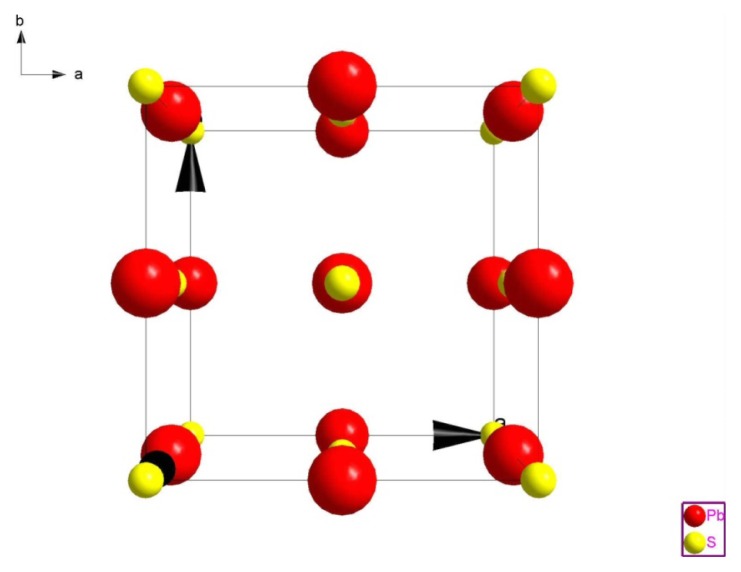
Structure of (PbS).

PbS has a sodium chloride-type structure, with a cubic system; space group (Fm3m) [9]. Noda *et al.* [10], obtained complete structural information as a function of temperature. Lead Pb is at position (0, 0, 0) and Sulfur S is at (1/2, 1/2, 1/2), as shown in Figure 2.

#### 2.1.3. Crystal Structure of Galenobismutite (PbS)(Bi_2_S_3_)

The crystal structure of Galenobismutite (PbS)(Bi_2_S_3_) was studied by several authors [8,10–13]. The lattice constants and space group are summarized in Table 1. The atomic coordinates are summarized in Table 2.

**Figure 3.**
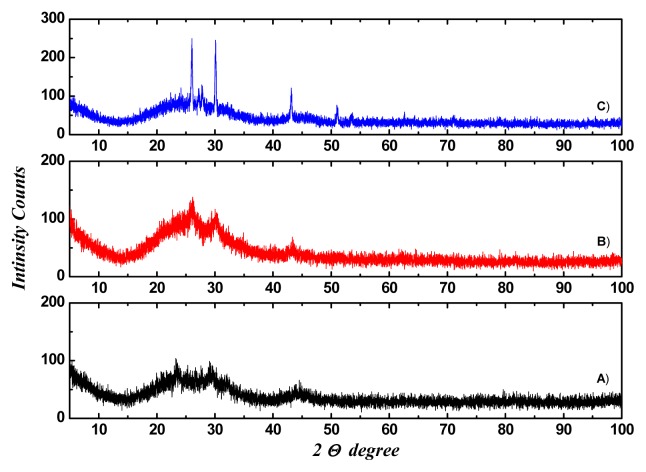
XRD of (Bi_2_S_3_)(PbS) thin films, (**A**) As-deposited; (**B**) Annealed at 623 K for 1 h; and (**C**) Annealed at 698 K for 4 h.

**Figure 4.**
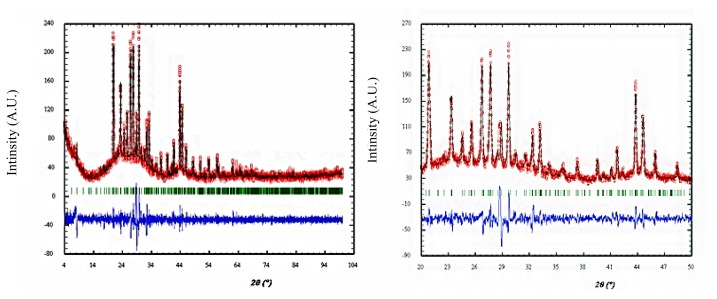
Lebail fit analysis of (Bi_2_S_3_)(PbS) thin films.

**Figure 5.**
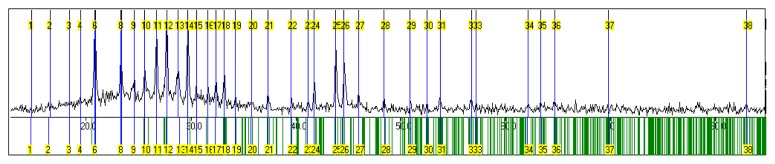
Measured reflections of (Bi_2_S_3_)(PbS) thin films.

**Figure 6.**
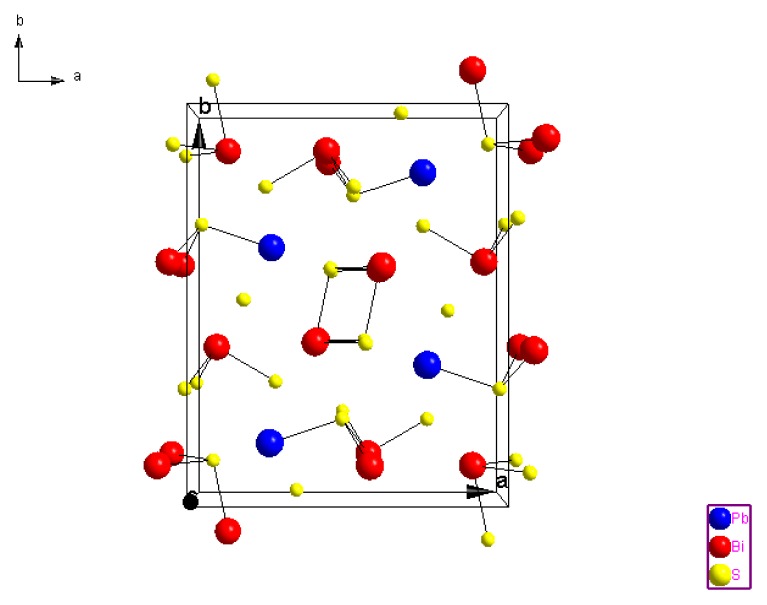
Structure of (Bi_2_S_3_)(PbS) thin films.

The structure of the Galinobisuitite (Bi_2_S_3_)(PbS) nanoparticulate thin films was calculated with the help of the Fullprof and Chekcell programs. Figure 3 shows the XRD patterns for the (Bi_2_S_3_)(PbS) films as-deposited and annealed at 623 K for 1 h, and annealed at 598 K for 4 h. The lattice parameters are illustrated in Table 3, a typical Lebail fit analysis is shown in Figure 4, and the indexing data are illustrated in Table 4. Typical peak matching is shown in Figure 5. Calculated crystal structure of (Bi_2_S_3_)(PbS) thin films is shown in Figure 6.

### 2.2. The Surface Morphology Studies

**Figure 7.**
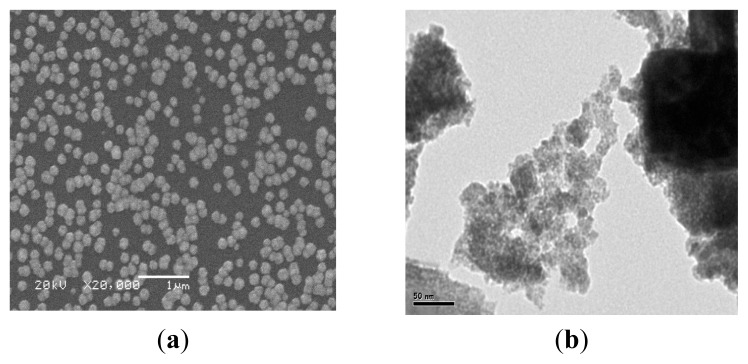
Surface morphology of (Bi_2_S_3_)(PbS) thin films, (**a**) SEM; (**b**) TEM.

The surface morphology of the (Bi_2_S_3_)(PbS) films is sensitive to compositions types, concentrations of the solutions mixtures, pH values, and both of the bath and annealing temperature These factors affect the structural morphology of the films [14]. Figure 7a shows that the small spherical nanograins, approximately 20–40 nm in size, were uniformly distributed over the smooth homogeneous background of the crystalline phase. The small grains are distributed rather distantly from each other, indicating the regular growth rate of the grains. Figure 7b shows the reduced grain density, indicating the noticeably smaller size of the grain. The RMS surface roughness is very slight.

### 2.3. The Optical Properties Studies

**Figure 8.**
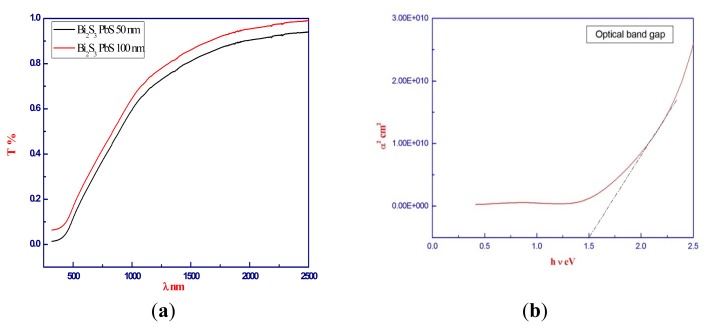
Optical properties of (Bi_2_S_3_)(PbS) thin films, (**a**) Typical transmission curves; (**b**) Optical band gap *E*_g_.

The typical transmission curves are shown in Figure 8a. The transmission samples showed gradual light absorption in a wide range of optical wavelengths (300–500 nm). The optical absorption edge of the films shifted toward a longer wavelength, which indicates that the optical band gap linearly decreased from 1.92 eV for Bi_2_S_3_, to 1.5 eV for Bi_2_S_3_ PbS.

Isomura *et al.* [14] and Susama *et al.* [15] calculated the optical constants and energy band gap of the films from the measured transmission using the following relation:

(1) α (h·ν)=1/d·ln 1/T

where *d* is the thickness of the films, and *T* is the transmission.

The optical band gap E_g_ were obtained from the linear portion of the (α^2^) *vs.* (h·ν) plot. An *E*_g_ value is obtained through the intersection of the straight line with the axis of the photon energy. In Figure 8b, the plot of (α^2^) *vs.* (h·ν) for direct transition of (Bi_2_S_3_)(PbS) films is shown. The confinement effect appears as a shift in edge of the absorption spectra and the absorption to lower wavelengths, possibly due to the decrease in the grain size and the decrease in the number of defects. It is clearly seen from the optical spectrum that an absorption edge shift toward a lower wavelength in the films occurs. Due to this effect, the *E*_g_ of the material increases as the size of the particle decreases. This property makes it an excellent candidate for opto-electronic applications in many fields, such as photography, IR detectors, solar absorbers, light emitting devices, and solar cells.

## 3. Experimental Section

### 3.1. Experimental Procedure

Some authors [8,16,17] reported the synthesis of (PbS)_6_(Bi_2_S_3_), (PbS)_3_(Bi_2_S_3_), (PbS)(Bi_2_S_3_), (PbS)_2_(Bi_2_S_3_), and (PbS)_4_(Bi_2_S_3_). They reported three ternary phases, designated as II, III, and IV, of the approximate compositions (PbS)_5_(Bi_2_S_3_), (PbS)_3_(Bi_2_S_3_), and (PbS)(Bi_2_S_3_). All these experiments were performed in closed, evacuated silica tubes in which a vapor phase was always present. Galinobisuitite (Bi_2_S_3_)(PbS) nanoparticle thin films were deposited on glass substrates (12 × 25 mm^2^) cleaned with detergent, degreased with trichloroethylene, acetone, and ethanol, rinsed with deionized water in an ultrasonic cleaner, and finally etched in a 10% HF solution immediately before use for the depositions [6]. Galinobisuitite (Bi_2_S_3_)(PbS) nanoparticle thin films were deposited from a solution; the chemical deposition bath was prepared according to the following procedures 1 M solution in distilled water, of bismuth nitrate-lead nitrate solution was complexed by the addition of triethanolamine. This was followed by the addition of 1 M thiourea, and 17 M ammonia solution and water. The basic overall reactions are based on Equations (2) and (3):

(2) $$2\text{Bi}{\left[\text{TEA}\right]}^{3+}+3\text{SC}{\left({\text{NH}}_{2}\right)}_{2}+6{\left[\text{OH}\right]}^{-}\to {\text{Bi}}_{2}{\text{S}}_{3}+3\text{OC}{\left({\text{NH}}_{2}\right)}_{2}+\text{TEA}+3{\text{H}}_{2}\text{O}$$

(3) $$\text{Pb}{\left[\text{TEA}\right]}^{2+}+\text{SC}{\left({\text{NH}}_{2}\right)}_{2}+2{\left[\text{OH}\right]}^{-}\to \text{PbS}+\text{OC}{\left({\text{NH}}_{2}\right)}_{2}+\text{TEA}+{\text{H}}_{2}\text{O}$$

To stop agglomeration, we used polyvinylpyrrollidone (PVP) as a capping agent after a few minutes of reaction, when the black precipitate was deposited on the glass substrate. The samples and holders were removed from the chemical bath after a deposition time of 60 min. The films obtained by this method, after ultrasonic cleaning, were smooth, uniform, and adherent. XRD measurements were made using Cu Kα radiation of wavelength λ = 0.15406 nm in the scan range 2θ = 4°–100°. Surface morphology was examined using a JEOL model JSM-6380 LA scanning electron microscope (SEM) (JEOL, Musashino, Tokyo, Japan). Surface roughness was measured using a DektakXT advanced system (Bruker, Billerica, MA, USA) with optional isolation pads. The formation of nanoparticles was confirmed using a transmission electron microscope (TEM, JEOL 1010, JEOL, Musashino, Tokyo, Japan). Optical transmission measurements were performed using a UV/VIS Jasco 7800 spectrophotometer (JASCO, Mary’s Court, Easton, PA, USA).

### 3.2. Measurment of Atomic Absorption

The powder sample (Bi_2_S_3_)(PbS) was digested by concentrated nitric acid (HNO_3_) and lead concentrations were determined according to the standard method [18]. For each analytical run, a calibration curve composed of a blank and three or more standards was within the method’s working range, and a replicate additional standard solution was analyzed after every sample. An external reference standard from Merck (MERCK, Darmstadt, Germany) and quality control samples from U.S.E.P.A (U.S.E.P.A, Washington, DC, USA) we are used to confirm the instrument’s lead concentration reading. The showed a slight difference between the desired and measured ratios of lead.

## 4. Conclusions

In summary, we prepared Galinobisuitite thin films of (Bi_2_S_3_)(PbS) using the chemical bath deposition technique (CBD). The grain size lies in the interval of 20–40 nm. We calculated the lattice parameters, space group, and crystal structure. The surface morphology showed that the small spherical nanograins were uniformly distributed over the smooth homogeneous background of the crystalline phase. Optical absorption spectra were quantified for the Galinobisuitite thin films of (Bi_2_S_3_)(PbS), in which the redshift of *E*_g_ was associated with the decrease in the average grain size. The direct band gap energy (*E*_g_) was in the range of 1.5 eV.

## Figures and Tables

**Table 1.** Lattice parameters of (PbS)(Bi_2_S_3_): A comparison.

Cell constant (Å)	Berry (1940)	Wickman (1951)	Itaka (1962)
a	11.72 (3)	11.65 (5)	11.79 (7)
b	14.52 (3)	14.49 (3)	14.59 (8)
c	4.07 (2)	4.08 (3)	4.10 (5)
Space group	(Pnam)	(Pna2)	(Pnam)

**Table 2.** Atomic parameters of Galenobismutite [9].

Axis	*X*	*Y*	*Z*
Pb	0.24792 (15)	0.65126 (10)	0.25000
Bi _(I)_	0.06750 (14)	0.39009 (10)	0.25000
Bi _(II)_	0.10427 (14)	0.90559 (11)	0.25000
S _(I)_	0.33072 (92)	0.01411 (69)	0.25000
S _(II)_	0.26091 (88)	0.29968 (74)	0.25000
S _(III)_	0.05499 (93)	0.09269 (71)	0.25000
S _(IV)_	0.01808 (97)	0.7119 (70)	0.25000

**Table 3.** Lattice parameters of (Bi_2_S_3_)(PbS).

*a*	*b*	*c*	α	β	γ	Volume	Space group
10.785	13.954	8.488	90	90	90	1277.52	P 222

**Table 4.** Indexing of peaks in (Bi_2_S_3_)(PbS).

No	*h*	*k*	*l*	2Θ(Obs)	2Θ(Calc)	Diff.	*d*-values
1	1	1	1	14.820	14.720	0.100	5.9776
2	0	2	1	16.540	16.446	0.094	5.3596
3	1	2	1	18.400	18.402	−0.002	4.8218
4	2	0	1	19.470	19.502	−0.032	4.5592
5	2	2	0	20.816	20.819	−0.003	4.2673
6	0	0	2	20.879	20.930	−0.051	4.2546
7	1	3	1	23.311	23.315	−0.004	3.8159
8	1	1	2	23.390	23.409	−0.019	3.8032
9	0	2	2	24.571	24.550	0.021	3.6230
10	3	1	0	25.597	25.587	0.010	3.4801
11	2	0	2	26.715	26.729	−0.014	3.3369
12	3	1	1	27.693	27.695	−0.002	3.2213
13	1	4	1	28.758	28.882	−0.124	3.1044
14	2	2	2	29.705	29.689	0.016	3.0075
15	2	4	0	30.527	30.520	0.007	2.9284
16	0	0	3	31.599	31.621	−0.022	2.8314
17	2	4	1	32.342	32.333	0.009	2.7681
18	1	5	0	33.148	33.158	−0.010	2.7026
19	0	2	3	34.204	34.196	0.008	2.6215
20	4	2	0	35.768	35.699	0.069	2.5104
21	2	4	2	37.310	37.300	0.010	2.4101
22	1	5	2	39.541	39.540	0.001	2.2791
23	3	1	3	41.176	41.109	0.067	2.1923
24	4	2	2	41.768	41.746	0.022	2.1630
25	5	1	1	43.788	43.785	0.003	2.0674
26	2	4	3	44.560	44.521	0.039	2.0334
27	2	0	4	45.973	45.958	0.015	1.9741
28	4	2	3	48.397	48.423	−0.026	1.8808
29	5	4	1	50.895	50.922	−0.027	1.7942
30	3	7	0	52.507	52.489	0.018	1.7428
31	6	2	1	53.735	53.756	−0.021	1.7058
32	4	2	4	56.747	56.760	−0.013	1.6222
33	1	7	3	57.162	57.167	−0.005	1.6115
34	4	6	3	62.122	62.077	0.045	1.4942
35	2	9	1	63.367	63.374	−0.007	1.4678
36	7	3	1	64.658	64.668	−0.010	1.4416
37	8	0	0	69.760	69.755	0.005	1.3481
38	6	4	5	82.940	82.989	−0.049	1.1641
